# A Homogeneous Hexagonal-Structured Polymer Electrolyte Framework for High-Performance Polymer-Based Lithium Batteries Applicable at Room Temperature

**DOI:** 10.3390/polym17131775

**Published:** 2025-06-26

**Authors:** Seungjin Lee, Changseong Kim, Suyeon Kim, Gyungmin Hwang, Deokhee Yun, Ilhyeon Cho, Changseop Kim, Joonhyeon Jeon

**Affiliations:** 1 Department of Advanced Battery Convergence Engineering, Dongguk University, Seoul 04620, Republic of Korea; 2 Division of Electronics and Electrical Engineering, Dongguk University, Seoul 04620, Republic of Korea

**Keywords:** single-ion conducting, gel polymer electrolytes, polymer electrolytes, anion receptor additive, lithium-ion transference number, electrochemical stability, polymer-based battery

## Abstract

In polymer-based lithium batteries, polymer electrolytes (PEs) exhibit limited ionic conductivity at room temperature (25 °C). To address this issue, this paper describes a hexagonal-structure-based single-ion conducting gel polymer electrolyte (*h*-SICGPE) framework with a robust and efficient cross-linked polymer network, applicable to polymer-based batteries even at 25 °C. The proposed cross-linked polymer network backbone of the *h*-SICGPE, as a semisolid-state thin film type, has the homogeneous honeycomb structure incorporating anion receptor(s) inside each of its hexagonal closed cells and is obtained by cross-linking between trimethylolpropane tris(3-mercaptopropionate) and poly(ethylene glycol) diacrylate in a newly synthesized anion–receptor solution. The excellent structural capability of the *h*-SICGPE incorporating Li^+^/TFSI^−^ can enhance ionic conductivity and electrochemical stability by suppressing crystallinity and expanding free volume. Further, the anion receptor in its free volume helps to effectively increase the lithium-ion transference number by immobilizing counter-anions. Experimental results demonstrate dramatically superior performance at 25 °C, such as ionic conductivity (2.46 mS cm^−1^), oxidative stability (4.9 V vs. Li/Li^+^), coulombic efficiency (97.65%), and capacity retention (88.3%). These results confirm the developed *h*-SICGPE as a promising polymer electrolyte for high-performance polymer-based lithium batteries operable at 25 °C.

## 1. Introduction

In polymer-based lithium batteries, polymer electrolytes (PEs) have been widely studied as promising alternatives to liquid electrolytes due to their enhanced safety, flexibility, and excellent electrode compatibility, making them attractive for next-generation polymer-based batteries [1]. However, the broad practical applications of PEs are constrained by inherent drawbacks such as low ionic conductivity and low lithium-ion transference number (LITN, $t_{Li⁺}$) at room temperature (25 °C). These limitations give rise to significant concentration polarization, increased internal impedance, and reduced rate performance [2,3]. Further, the poor ionic conductivity primarily stems from polymer crystallinity associated with high glass transition temperature (T_g_), which restricts polymer chain segmental motion and impedes ion mobility [4,5,6]. Conventional PEs function as dual-ion conductors, allowing both lithium-ions and counter-anions in opposite directions during charge–discharge cycles [7]. Since lithium-ions tend to strongly coordinate with Lewis–base sites on polymer chains, their mobility is substantially lower compared to more mobile counter-anions, aggravating concentration polarization [8,9]. This polarization promotes uneven lithium deposition and dendritic formation, adversely affecting battery lifespan and safety. Addressing these critical challenges is therefore essential to fully realize practical, high-performance polymer batteries.

Among various polymer electrolytes, polyethylene oxide (PEO) electrolytes are particularly prominent due to their superior strong lithium-ion solvating capability and excellent compatibility with various lithium salts [10,11]. Despite these advantages, PEO electrolytes suffer from high crystallinity at 25 °C, resulting in insufficient ionic conductivity and poor battery performance under practical conditions [12]. Recently, Li et al. [13] reported that employing cross-linked polymer structures, known as network polymers, leads to reduced crystallinity and improved oxidative stability, while simultaneously expanding amorphous regions advantageous for ionic conduction. Deng et al. [14] reported a cross-linked network structure integrating plasticizers to form gel polymer electrolytes (GPEs) which significantly enhances ionic conductivity by facilitating lithium salt dissociation and increasing segmental mobility. However, these previous studies have a serious drawback of providing low LITNs caused by forming the strong coordination between lithium-ions and polymer chains, which is attributed to accelerating lithium dendrite growth during cycling [15,16,17]. Dai et al. [18] highlighted the effectiveness of boron-based anion receptors incorporated within polymer matrices in enhancing LITNs. Particularly, boron atoms in these anion receptors possess an empty *p*-orbital capable of trapping counter-anions through π-interactions and forming stable sp^3^ π-complexes that significantly limit anion mobility and thus facilitate lithium-ion transport [3,19,20]. Ma et al. [21] introduced an allylboronic acid pinacol ester (AAPE) with strong Lewis acid properties into PE systems to efficiently immobilize counter-anions, highlighting the simplicity, versatility, and effectiveness of this synthetic strategy [22,23,24]. The approaches of ref. [18,21] collectively represent promising strategies for designing advanced single-ion conducting gel polymer electrolytes (SICGPEs) able to significantly enhance LITNs [25,26,27,28]. Nevertheless, there still exist problems in developing PEs capable of simultaneously delivering both high ionic conductivity and high LITNs at 25 °C, requiring novel electrolyte systems that effectively balance these critical properties.

To address these challenges, this paper presents a state-of-the-art hexagonal-structure-based (*h*-) SICGPE framework by dramatically forming a cross-linked polymer network structure that possesses anion receptor groups within the polymers. The proposed cross-linked polymer network backbone of the *h*-SICGPE has a hexagonal-cell structure as a solid-state din film type and is obtained by cross-linking between trimethylolpropane tris(3-mercaptopropionate) (TMPMP) and poly(ethylene glycol) diacrylate (PEGDA, average Mn 700) in a newly synthesized anion receptor solution, “pentaerythritol tetrakis[3-({3-[(4,4,5,5-tetramethyl-1,3,2-dioxabolan-2-yl)methyl]propyl}sulfanyl)-3-oxopropanoate] (PTO)”. The PTO, as a solution for the polymerization of cross-linking two monomers and the deposition of anion receptors within the polymers, is obtained from thiol-ene click reaction between pentaerythritol tetrakis(3-mercaptopropionate) (PTM) and allylboronic acid pinacol ester (AAPE). Finally, the *h*-SICGPEs are obtained by soaking the solid-state polymer film in the plasticizer solution including lithium salt. In the proposed *h*-SICGPE framework, the cross-linked TMPMP-PEGDA polymer (TPP) network ensures a high stability and excellent structural capability by suppressing crystallinity and expanding the amorphous region (with anion receptor) in the free volume of the TPP network, resulting in a significant enhancement of ionic conductivity even at 25 °C by strongly and effectively immobilizing counter-anions. These celebrated synergistic effects are supported by strong Lewis acid–base interactions and an optimized amorphous network architecture. The superior electrochemical properties are demonstrated through electrochemical impedance spectroscopy (EIS), chronoamperometry (CA), linear sweep voltammetry (LSV), lithium plating/stripping cycle tests, and galvanostatic cycling tests. As a result, the developed *h*-SICGPE achieves remarkably high performance at 25 °C, such as high ionic conductivity (~2.46 mS cm^−1^, 581.6 times higher than PEO), apparent LITNs ($t_{Li⁺}$ = 0.805), high oxidative stability (~4.9 V vs. Li/Li^+^), excellent coulombic efficiency (95.8%), and high capacity retention (88.3%) conditions, offering a promising solution for next-generation polymer-based batteries.

**Motivation and Design Strategy of *h*-SICGPE:**

**Scheme 1:**
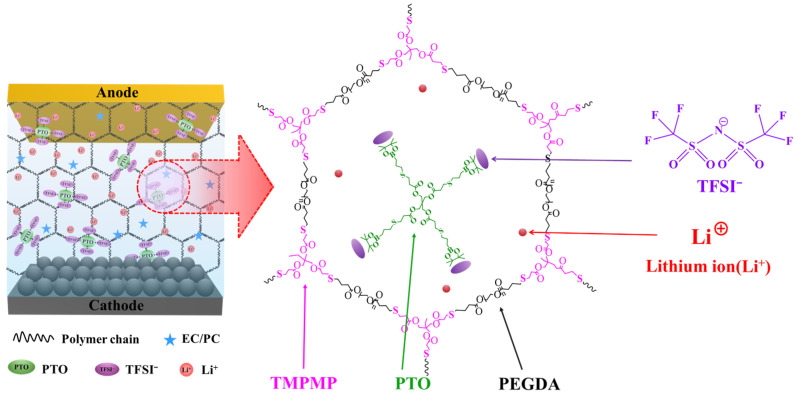
The summarized chemical composition and network structure of *h*-SICGPE.

The key highlight of this study is to overcome the long-standing tradeoff between ionic conductivity and LITN in PEs operating at 25 °C. Conventional dual-ion conducting systems, including those based on PEO, often suffer from low LITNs [29]. This is primarily due to the high mobility of counter-anions, along with the strong coordination between lithium ions and ether oxygen atoms. These two factors cause concentration polarization and thereby promote the growth of lithium dendrites. To address these challenges, this paper deals with a novel *h*-SICGPE framework of a cross-linked polymer network backbone possessing anion receptors in its large free volumes, attributed to the long chain of PEGDA, where the *h*-SICGPE’s hexagonal structure is formed by the thiol-ene click reaction between PEGDA and TMPMP monomers in the PTO solution. The *h*-SICGPE, able to assure such remarkable performance in 25 °C, can be successfully achieved by incorporating PTO (anion receptor) into the free volume of the cross-linked TPP network. The PTO with a robust cross structure is a boronate-functionalized anion receptor capable of efficiently and effectively suppressing the mobility of anionic species and enhancing LITNs in the *h*-SICGPE, which strongly traps counter-anions via Lewis acid–base interactions and π-complexation. Further, the cross-linked TPP network with a homogeneous hexagonal cell structure significantly enhances ionic conductivity and provides robust mechanical integrity by strongly suppressing crystallinity and expanding the amorphous region. Notably, the TPP polymer matrix incorporates both ether and ester functionalities, allowing for balanced lithium coordination and improved segmental mobility [30]. The synergistic effect of incorporating the PTO receptor in the hexagonal-cell structure of the cross-linked TPP network offers a viable pathway toward realizing SICGPEs with outstanding electrochemical performance and stability at 25 °C. Scheme 1 shows the summarized chemical composition and network of the *h*-SICGPE.

## 2. Materials and Methods

### 2.1. h-SICGPE Preparation

#### 2.1.1. Materials

For synthesizing *h*-SICGPE, nine kinds of materials were used as follows: Pentaerythritol tetrakis(3-mercaptopropionate) (PTM, Sigma-Aldrich, St. Louis, MO, USA), allylboronic acid pinacol ester (AAPE, Sigma-Aldrich), trimethylolpropane tris(3-mercaptopropionate) (TMPMP, Sigma-Aldrich), poly(ethylene glycol) diacrylate average Mn 700 (PEGDA, Sigma-Aldrich), 2,2-dimethoxy-2-phenylacetophenone (DMPA, Sigma-Aldrich), acetonitrile (ACN, SAMCHUN CHEMICAL, Seoul, Republic of Korea), ethylene carbonate (EC, TCI, Tokyo, Japan), propylene carbonate (PC, TCI), and lithium bis(trifluoromethanesulfonyl)imide (LiTFSI, MTI, Richmond, CA, USA).

#### 2.1.2. PTO Preparation: Boronate-Functionalized Anion Receptor

**Figure 1:**
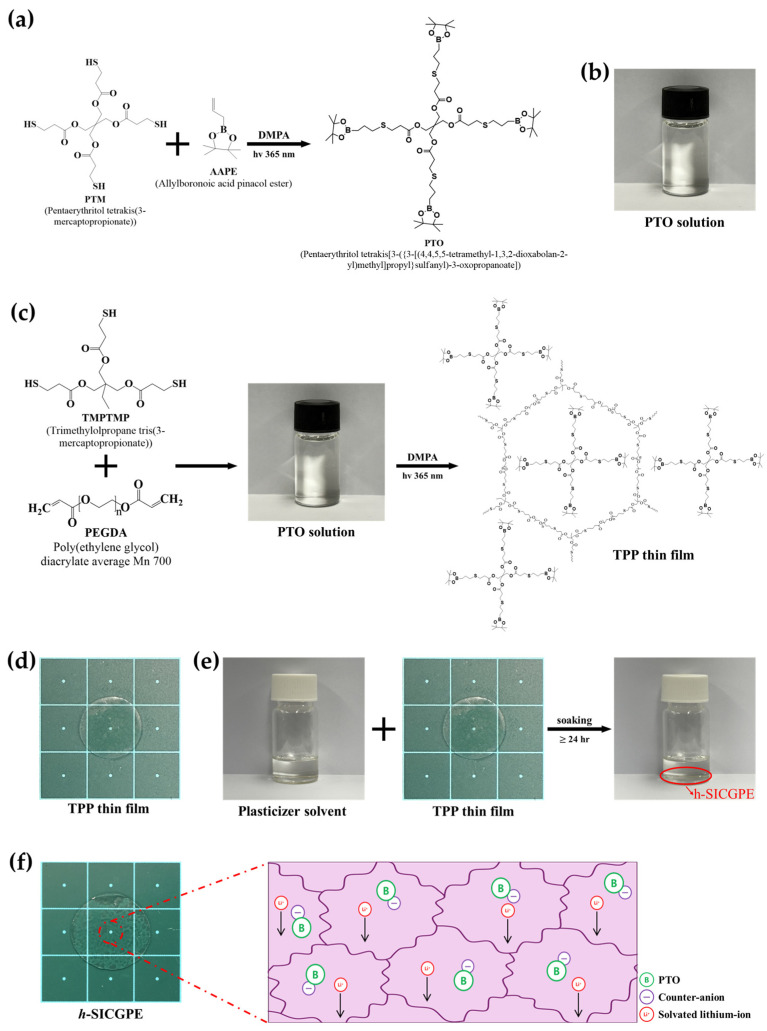
(**a**) Synthesis schematic and (**b**) photograph of synthesized PTO solution, (**c**) synthesis schematic and (**d**) photograph of cross-linked TPP network backbone (solid-state polymer film), (**e**) soaking process and (**f**) *h*-SICGPE’s photograph and schematic illustration.

To prepare the boronate-functionalized anion receptor solution, PTM (6.075 mmol), AAPE (24.3 mmol), and DMPA (0.243 mmol) were dissolved in 30 mL of ACN under stirring. The photo-initiator DMPA was used to initiate the thiol-ene click reaction between PTM and AAPE. Once the solution was thoroughly mixed at 25 °C, it was subjected to UV irradiation lamp (365 nm) for 10 min. Figure 1a shows synthesis schematic and chemical structure of PTO solution developed in this work. For PTO preparation, the DMPA (photo-initiator) was added into PTM and AAPE and exposed to a 365 nm ultraviolet light. Then, photodecomposition of DMPA in the mixture initiates a thiol-ene click reaction. This reaction continues until all reactive monomers are fully consumed, benefiting from rapid reaction kinetics, high conversion efficiency, and excellent selectivity [31]. Following the completion of the reaction, a transparent solution containing PTO is obtained as shown in Figure 1b.

#### 2.1.3. Cross-Linked TPP Network Polymer Polymerization

For the polymerization of the cross-linked TPP network backbone, TMPMP (2 mmol) and PEGDA (average Mn 700) (3 mmol) were added in the prepared PTO solution of 3 mL, which is the boronate-functionalized anion receptor solution synthesized in Section 2.1.2. This is to incorporate PTO into the cross-linked TPP network. An additional DMPA (0.056 mmol) was also used as a photo-initiator, and the mixture was stirred thoroughly to obtain a well-blended solution. Subsequently, 1 mL of the resulting solution was cast into a polytetrafluoroethylene (PTFE) mold and exposed to a 365 nm UV irradiation lamp at 25 °C for 10 min to initiate thiol-ene click photopolymerization. The cured polymer was then placed in a vacuum oven and dried for more than 24 h to remove residual solvents. Figure 1c presents the synthetic illustration and hexagonal-cell structure of the TPP network backbone. When the PTO solution containing TMPMP, PEGDA (average Mn 700), and DMPA (as photo-initiator) is exposed to 365 nm ultraviolet light, thiol-ene click photopolymerization is initiated by the activated DMPA in the PTO solution. Then, three-dimensional (3D) polymer network is formed through cross-linking between TMPMP and PEGDA (average Mn 700) in the PTO solution, and consequently, there exist anion receptors in free volumes of cross-linked polymer network structure. Figure 1d shows the final solid-state polymer film obtained after photopolymerization and vacuum drying, resulting in a mechanically robust electrolyte structure.

#### 2.1.4. h-SICGPE Preparation: Synthesizing the Gel Polymer Electrolyte

The plasticizer solvent was prepared by mixing EC and PC at a 3:1 weight ratio [32], followed by the addition of LiTFSI to reach a 1.2 M concentration. After thorough stirring, the solid-state TPP thin film of Figure 1d was immersed in the solution and soaked for more than 24 h to allow sufficient uptake. Figure 1e presents a schematic diagram of the *h*-SICGPE. Following immersion of the prepared TPP thin film in the plasticizer solution, lithium-ions and counter-anions are incorporated into the TPP hexagonal-cell matrix for establishing the ionic environment depicted in the figure. This synthesis process suggests that the designed *h*-SICGPE has a robust and effective structure containing anion receptors and Li^+^/TFSI^−^ in free volumes (hexagonal cells) of cross-linked TPP network structure. Figure 1f displays the final *h*-SICGPE obtained after the soaking process, indicating successful electrolyte uptake and the formation of a uniform gel electrolyte phase.

### 2.2. FT-IR Spectra Analysis

The evaluation of the developed *h*-SICGPE was demonstrated through analyzing a Fourier transform infrared (FT-IR) spectra of the PTO solution and the TPP film. For this purpose, FT-IR spectrometer (Smiths Detection IdentifyIR^®^, Hemel Hempstead, UK) was used, and the spectra results were recorded in the range of 4000–650 cm^−1^ with 256 scans per sample.

### 2.3. Analysis of Thermal Characteristics

Thermal stability of the prepared *h*-SICGPEs was analyzed by using thermo gravimetric analyzer (TGA) (SCINCO pyris TGA/N-1000, Twin Lakes, WI, USA). The TGA measurements were performed from 25 °C to 600 °C at a heating rate of 10.00 °C/min under a nitrogen atmosphere. The thermal transitions of *h*-SICGPE were also characterized by using differential scanning calorimeter (DSC) (Perkin Elmer Jade DSC, Shelton, CT, USA) in the heat range of 0 °C to 100 °C at a scan rate of 10.00 °C/min.

### 2.4. Electrochemical Analysis

To measure the ionic conductivity of *h*-SICGPEs, the electrochemical impedance spectroscopy (EIS) was conducted using ZIVE MP1 (WonATech, Seoul, Republic of Korea). The measurements were carried out in a stainless steel (SS)|*h*-SICGPE|SS symmetric cell configuration, with an applied AC voltage of 10 mV amplitude over a frequency range from 1 MHz to 0.1 Hz. Impedances of the proposed *h*-SICGPEs were measured at 25 °C, 30 °C, 40 °C, 50 °C, and 60 °C. Then, the ionic conductivity (σ) was calculated using the following Equation (1):

(1) $$\sigma =\frac{l}{RA}$$

where *A*, *R*, and l denote the electrode active area, bulk electrolyte resistance, and the *h*-SICGPE thickness, respectively. The electrochemical stability window (ESW) of *h*-SICGPE was evaluated by LSV measurement at a scan rate of 10 mV s^−1^ under a voltage sweep from 2.0 to −0.2 V and 2.0 to 6.0 V (vs. Li/Li^+^) at 25 and 60 °C, then SS|*h*-SICGPE|Li asymmetric cell was also used. In addition, to evaluate LITNs, chronoamperometry (CA) and EIS measurements were conducted using Li|*h*-SICGPE|Li symmetric cells at constant potential of 10 mV. The transference numbers were then calculated using the following Bruce–Vincent method’s Equation (2) [33]:

(2) $$t_{{Li}^{+}}=\frac{I_{S}(∆V-I_{0}R_{0})}{I_{0}(∆V-I_{S}R_{S})}$$

where *I_s_*, *I*_0_, ∆*V*, and *R*_0_/*R_s_* denote steady-state current, initial current, polarization potential, and interfacial resistances before/after the steady-state, respectively. To demonstrate interfacial stability during repeated lithium deposition and dissolution, galvanostatic lithium plating/stripping cycle tests were performed using WHW-25L-S-16CH (NEWARE, Shenzhen, China) battery testing system. Li|*h*-SICGPE|Li symmetric cells were cycled at a constant current density of 0.1, 0.2, 0.5, 1.0 mA cm^−2^ for one hour per cycle.

### 2.5. Galvanostatic Charge/Discharge Cell Tests

Coin cell (CR2032-type) with a configuration of LiFePO_4_ (LFP)|*h*-SICGPE|Li asymmetric cell was assembled, which consists of LFP, lithium metal, and *h*-SICGPE as cathode, counter electrode, and electrolyte, respectively. Galvanostatic charge/discharge cell tests were performed at 25 °C using WHW-25L-S-16CH (NEWARE, China) battery testing system. The cells were cycled at a current rate of 0.5 C within a voltage window of 2.50–3.65 V.

## 3. Results and Discussion

### 3.1. Chemical Bonds and Structural Analysis of PTO Solution and TPP Thin Film

**Figure 2:**
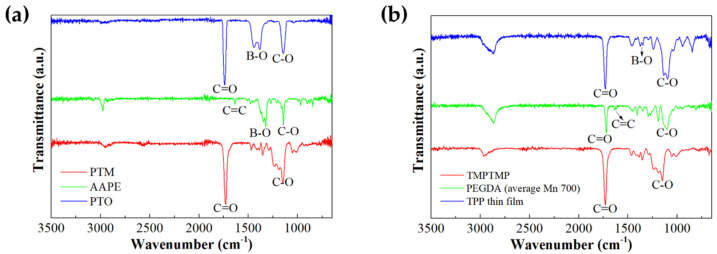
FT-IR Spectra analysis of (**a**) PTO solution and (**b**) cross-linked TPP film of *h*-SICGPE.

To analyze the developed *h*-SICGPE structure of the cross-linked polymer network incorporating anion receptors, Figure 2a,b show the FT-IR spectra of the synthesized PTO solution and solid-state TPP thin film (with thickness of 245 μm) mentioned in Figure 1a,c. In the (green line) spectrum of the AAPE used for synthesizing the PTO solution (i.e., anion receptor), the C=C stretching peaks appear near 1635 cm^−1^, and disappear in the (blue line) spectrum of the synthesized PTO. This means that the C=C bonds in AAPE are completely consumed in the thiol-ene click reaction [34,35,36,37]. Further, the B–O, C=O, and C–O stretching peaks originating from AAPE and PTM (red line) also appear in the PTO spectrum, confirming the successful formation of the desired structure [38,39,40]. In addition, the C=C stretching band near 1635 cm^−1^, observed in the (green line) spectrum of PEGDA (average Mn 700), disappears entirely in the (blue line) spectrum of the synthesized TPP thin film of Figure 1d, indicating full conversion of the acrylate groups in the photo-cross-linking process. Moreover, the clear presence of the characteristic B–O stretching band near 1350 cm^−1^ in the TPP thin film spectrum further confirms the successful incorporation of PTO into the polymer network. Furthermore, in the TPP thin film spectrum, the appearance of C=O and C–O bands observed from both TMPMP (red line) and PEGDA indicates the successful formation of cross-linking bonds between two monomers. Consequently, these FT-IR results can clearly assure the successful incorporation of anion receptors in the cross-linked TPP network.

### 3.2. Thermal Characteristics Analysis of h-SICGPE

**Figure 3:**
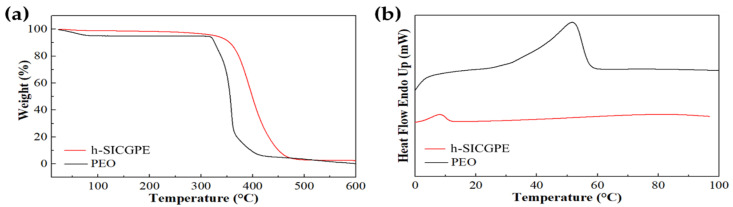
TGA and DSC analysis of *h*-SICGPE at a heating rate of 10.00 °C/min. (**a**) TGA measurements under nitrogen atmosphere from 0 to 600 °C and (**b**) DSC recorded from 0 °C to 100 °C.

To evaluate the thermal properties of the proposed *h*-SICGPE, TGA and DSC analysis were performed and compared to typically well-known PEO electrolytes [41]. Figure 3a shows TGA curves demonstrating the thermal decomposition behaviors of both polymer electrolytes. The conventional PEO electrolyte sharply decomposes around 320 °C, while the designed *h*-SICGPE begins relatively gentle thermal degradation at approximately 363 °C. The *h*-SICGPE exhibits an enhanced thermal stability approximately 43 °C higher than PEO, which highlights its excellent thermal robustness suitable for practical battery applications. Figure 3b depicts DSC thermograms illustrating the thermal transition characteristics of the *h*-SICGPE. The melting temperature (T_m_) of the *h*-SICGPE is significantly lower (8–10 °C) compared to the PEO electrolyte (~51 °C), which is attributed to the formation of the large (hexagonal) free volume and amorphous region of the polymer matrix for a greater ion transfer path in the cross-linked TPP network. Considering that the melting temperature typically appears higher than the glass transition temperature (T_g_) and crystallization temperature (T_c_), it can be inferred that both the T_g_ and T_c_ of the *h*-SICGPE are well below 25 °C. These low-temperature transitions (T_g_, T_c_, and T_m_) below 25 °C ensure sufficient chain mobility and continuous ion transport pathways between anodes and cathodes of polymer-based battery cells. Importantly, no abnormal weight loss and thermal events appear in both analysis curves of the *h*-SICGPE, indicating complete conversion of monomers to the PTO and cross-linked TPP network described in Section 2.1.

### 3.3. Effect of PTO-TPP Incorporation on LITN Enhancement

**Figure 4:**
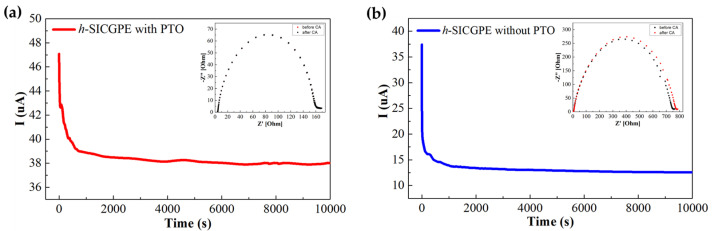
CA results and Nyquist plots (the insets) before/after polarization of Li|*h*-SICGPE|Li symmetric cells with (**a**) *h*-SICGPE, (**b**) *h*-SICGPE without PTO.

Figure 4a illustrates the CA results of the *h*-SICGPE incorporating PTOs (anion receptors) into the cross-linked TPP network, where the LITN is calculated using Bruce–Vincent Equation (2) based on current and impedance measurements before and after polarization [33]. The calculated LITN value is 0.805 significantly higher than LITN values reported for similar gel polymer electrolytes [42,43]. From the Nyquist plot shown in the inset of Figure 4a, it can be seen that the interfacial resistance between *h*-SICGPE and the Li electrode is very slightly increased from 4.487 to 4.491 Ω after CA measurement, indicating a stable and robust interfacial interface of the Li|*h*-SICGPE|Li symmetric cell. This stable interfacial behavior ensures an excellent electrochemical capability of the *h*-SICGPE to sustain strong concentration gradient recovery and mechanical integrity for a given potential. To clearly demonstrate the effectiveness of PTO-TPP network incorporation, as compared to an *h*-SICGPE without PTO (LITN = 0.333), shown in Figure 4b, the *h*-SICGPE with PTO exhibits an approximately 2.4 times higher LITN. This enhancement aligns well with the proposed mechanism wherein the boron atom’s Lewis acid sites in PTO immobilize counter-anions, thereby facilitating lithium-ion transport. This highlights the significant improvement in lithium-ion transport efficiency achieved through the rational design and synthesis of the PTO-integrated *h*-SICGPE in this work.

### 3.4. Electrochemical Performances of h-SICGPE

**Figure 5:**
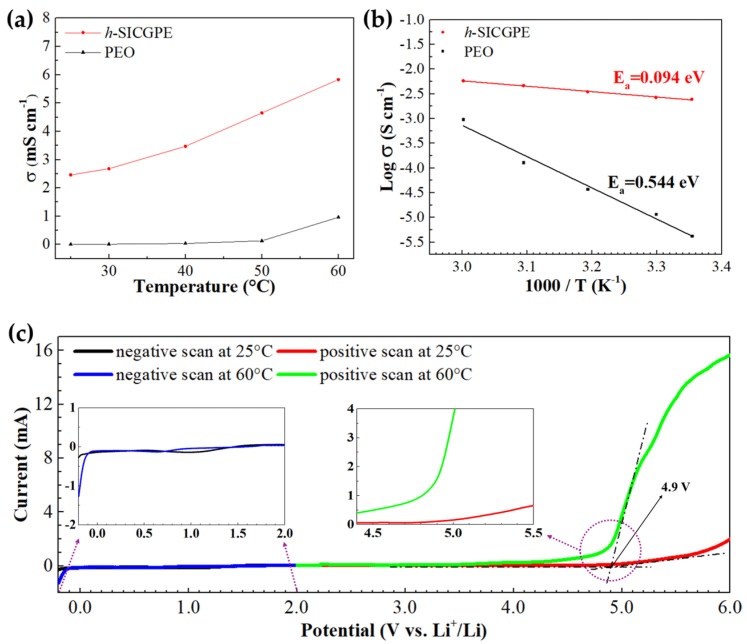
(**a**) Ionic conductivities and (**b**) Arrhenius plot of ionic conductivities of *h*-SICGPE and PEO electrolyte at different temperatures; (**c**) LSV curves of *h*-SICGPE at 25 and 60 °C.

Figure 5a shows that the designed *h*-SICGPE leads to dramatically high conductivities of 2.46 and 5.83 ± 0.03 mS cm^−1^ at 25 to 60 °C, respectively, higher than those of similar gel polymer electrolytes [42,43]. This is attributed to improved segmental motion and ion mobility due to polymer chain cross-linking and LiTFSI plasticizer soaking during the synthesis of the *h*-SICGPE. It can be seen that the proposed *h*-SICGPE at 25 °C exhibits 582 times higher ionic conductivity than conventional the PEO electrolyte of 4.23 ± 0.07 × 10^−3^ mS cm^−1^. These results can be clearly confirmed through the Arrhenius fitting curves of Figure 4b taken from Figure 5a. Figure 5b displays Arrhenius plots of ionic conductivity (versus the reciprocal of temperature 1/T), indicating that the *h*-SICGPE has a higher correlation coefficient (R^2^ = 0.9951) and lower activation energy (E_a_ = 0.094 eV) than PEO (R^2^ = 0.9543, E_a_ = 0.544). This implies that the ion transport mechanism is more effectively and efficiently enhanced in the *h*-SICGPE than PEO, because the increased free volume of the polymer matrix (by the structural superiority of cross-linked TPP network) increases the polymer chain’s mobility.

Figure 5c depicts the electrochemical stability of the *h*-SICGPE through LSV measurements at 25 and 60 °C, where the LSV scan was conducted separately from 2.0 V to −0.2 V (vs. Li/Li^+^) and 2.0 V to 6.0 V at a scan rate of 10 mV s^−1^, respectively [44]. It can be seen that both current oxidation peaks in the positive scan curves at 25 (red line) and 60 °C (green line) are detected at approximately 4.9 V (as measured by the tangent method), which is more than the maximum operational voltage 4.5 V of commercially available high-energy-density cathode materials, such as LiCoO_2_, NCM, and NCA. The current reduction peaks in the negative scan curves at 25 (black line) and 60 °C (blue line) begin at around 1.0 and 0.5 V, respectively, and their low reduction currents are shown in the lithium plating process region (−0.2 to 0 V). These results demonstrate that the *h*-SICGPE with a high potential of 4.9 V at 25 °C not only possesses high oxidation stability for the high-voltage operation of polymer batteries, but also exhibits superior solid electrolyte interphase (SEI) stability for practical cell operation.

### 3.5. Cell Tests

**Figure 6:**
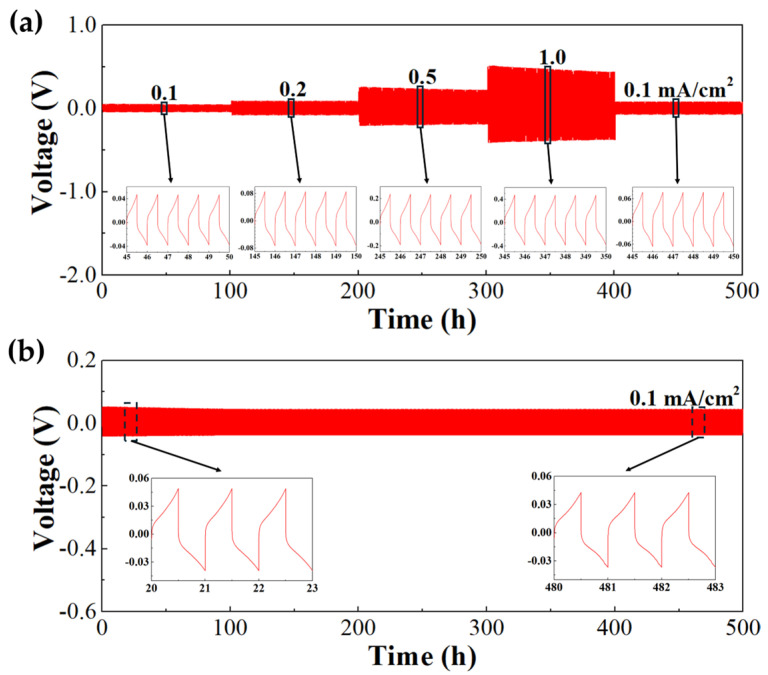
(**a**) Voltage-time profiles of the Li|*h*-SICGPE|Li symmetric cells at 25 °C for (**a**) 0.1, 0.2, 0.5, and 1.0 mA cm^−2^ (inset: middle five cycles) and (**b**) 0.1 mA cm^−2^ (inset: voltage profiles at the ranges of 20–23 h and 480–483 h).

Figure 6a presents voltage profiles of lithium plating/stripping cycle tests using a symmetric Li|*h*-SICGPE|Li symmetric cell configuration, which were cycled at increasing current densities of 0.1, 0.2, 0.5, and 1.0 mA cm^−2^, followed by a return to 0.1 mA cm^−2^. The *h*-SICGPE exhibits stable voltage profiles with consistent overpotentials, demonstrating robust interfacial stability and excellent reversibility of lithium plating/stripping. Further, the inset voltage profiles (middle five cycles at each current density) confirm superior reversibility, highlighting no polarization changes associated with increased current densities. In the long-term symmetric cell cycling at 0.1 mA cm^−2^ (Figure 6b), the *h*-SICGPE shows a significantly stable and reversible (lithium plating/stripping) behavior continued for 500 h without short circuiting, indicating excellent interfacial compatibility with lithium metal electrodes. This cycling stability, which is ascribed to the suppression of lithium dendrite formation by the high LITN ($t_{{Li}^{+}}$ = 0.805) of the *h*-SICGPE, is because PTOs (anion receptors) incorporated in the cross-linked TPP polymers interact with mobile anions via Lewis acid–base interactions, effectively immobilizing counter-anions [45].

These results suggest that the excellent structural superiority of the *h*-SICGPE facilitates uniform lithium-ion flux and smooth deposition behavior even at 25 °C.

**Figure 7:**
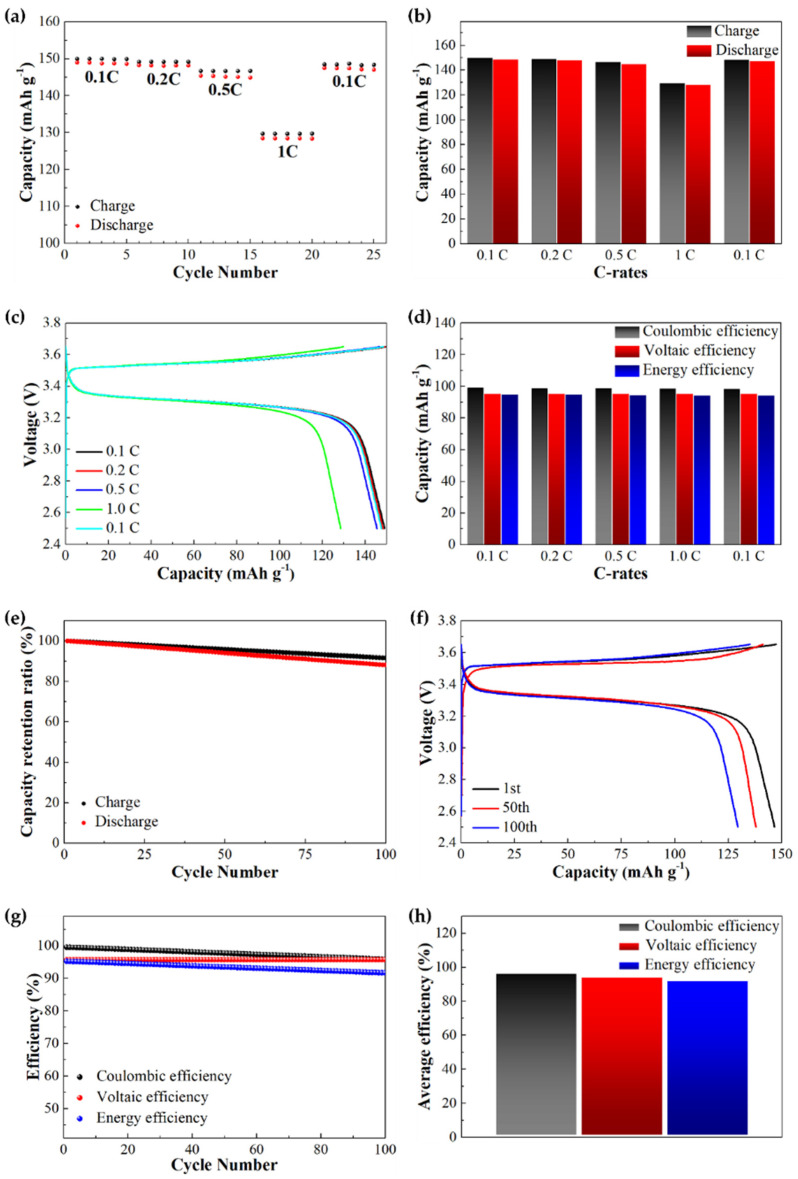
Performance profiles of the LFP|*h*-SICGPE|Li cell: (**a**) C-rate capability, (**b**) average capacity comparisons, (**c**) charge/discharge voltage profiles of the 1st cycle, and (**d**) average efficiencies at 0.1, 0.2, 0.5, 1.0, and 0.1C for five cycles each; (**e**) capacity retention rate for 100 cycles, and (**f**) charge/discharge voltage profiles at 1st, 50th, 100th cycle, (**g**) efficiency profiles for 100 cycles and (**h**) average efficiencies for 100 cycles.

To further assess full-cell performance, LFP|*h*-SICGPE|Li asymmetric cells were assembled and tested under various galvanostatic charge/discharge conditions at 25 °C. Figure 7a–d display the cycling performance at 0.1, 0.2, 0.5, and 1.0 C-rates with five cycles each, indicating that the charge and discharge capacities show typical rate-dependent behavior. When returning to 0.1 C, the *h*-SICGPE delivers a highly reversible discharge capacity of 147.3 mAh g^−1^ which is only a 1.0% discharge capacity loss (vs. 148.8 mAh g^−1^ at the initial 0.1 C). Based on these results, a 0.5 C-rate (yields the discharge capacity of 145.2 mAh g^−1^) is selected for the long-term cycling to reflect practical operating conditions while maintaining moderate electrochemical stress [46]. Figure 7e–h depict the cycling performances for 100 cycles at 0.5 C determined for the electrochemical stress-test of the *h*-SICGPE. In general, the capacity loss is because as the C-rate increases from 0.1 to 1.0 C, the available capacity decreases due to the resistance increase in the polymer battery and the chemical reaction rate retardation of the active species [47]. Nevertheless, the most surprising thing to note is that, despite the room temperature cycling process, the *h*-SICGEP exhibits a significantly excellent charge and discharge capacity retention. As a result, Figure 7e reveals the charge and discharge capacity retention ratios compared to the initial charge and discharge capacities for 100 cycles at 0.5 C. The results show that the *h*-SICGPE leads to significantly outstanding charge and discharge capacity retentions averaging 95.81 and 94.05%, with initial charge and discharge capacities of 147.5 and 146.7 mAh g^−1^, respectively, resulting in a highly stable and reversible reaction in the LFP|*h*-SICGPE|Li asymmetric cell. This implies that the capacity loss during the long-term cycling is almost negligible, and the *h*-SICGPE shows excellent ionic conductivity (2.46 mS cm^−1^ at 25 °C). The most striking effect of the *h*-SICGPE network structure on the charge and discharge capacities can be further confirmed by comparing the 1st, 50th, and 100th cycle curves described in Figure 7f, indicating that the 100th cycle shows slight charge and discharge capacity losses of only 8.3 and 11.7% vs. the 1st cycle. It also appears that the average charge and discharge voltages of 3.560 and 3.254 V at the 100th cycle are nearly similar to the 3.561 and 3.255 V of the 1st cycle, respectively, implying stable interfacial contact and effective lithium-ion transport over time. These results explicitly demonstrate that the *h*-SICGPE is electrochemically stable and structurally durable for long-term cycling. Figure 7g depicts the performance of the CE, VE, and EE for 100 cycles, resulting in average efficiencies of 97.65 (CE), 95.65 (VE), and 93.40 (EE) %, respectively, as indicated in Figure 7h. The efficiency profiles show that during 100 cycles, the CE slightly decreases from 99.50 to 95.80% and surprisingly, the VE keeps very stable (maintaining 95.65%), implying that there are no energy losses that occur during the charging process. This is because the *h*-SICGEP leads to robust interfacial stability and excellent reversibility of lithium plating/stripping, as demonstrated earlier.

These results explicitly demonstrate that the *h*-SICGPE, which has superb Li^+^ kinetics and reversibility with high polymer-electrolyte utilization at 25 °C, is electrochemically stable and structurally durable even for long-term cycling at room temperature.

## 4. Conclusions

In polymer-based lithium batteries, the utilization of polymer electrolytes at room temperature (25 °C) should overcome various problems including low ionic conductivity due to crystallization and high interfacial resistance between the solid polymer electrolyte and the electrode. To address these challenges, this study has presented a novel *h*-SICGPE framework as a polymer electrolyte of film type, applicable to polymer-based batteries even at room temperature. A newly developed cross-linked TPP network backbone of the proposed *h*-SICGPE has the homogeneous honeycomb structure of incorporating anion receptors inside each of its hexagonal closed-cells. The experimental results have demonstrated that the proposed *h*-SICGPE incorporating Li^+^/TFSI^−^ exhibits high ionic conductivity and electrochemical stability by suppressing crystallinity and expanding free volume. Further, it has been shown that the anion receptor in its free volume helps to effectively increase the lithium-ion transference number by immobilizing counter-anions. Long-term cycling tests using LFP|*h*-SICGPE|Li asymmetric cells at room temperature have shown the robust interfacial stability and excellent reversibility of lithium plating/stripping.

Consequently, this study provides new insight into the *h*-SICGPE with a robust and efficient cross-linked TPP network structure incorporating anion receptors, as a promising gel polymer electrolyte for high-performance polymer-based battery applications.

## Data Availability

The original contributions presented in this study are included in the article. Further inquiries can be directed to the corresponding author.
